# LINC00460 Promotes Cutaneous Squamous Cell Carcinoma Progression Through Stabilizing ELAVL1 Protein

**DOI:** 10.1007/s12033-022-00631-9

**Published:** 2022-12-13

**Authors:** Chunli Xue, Zuxian Yang, Ben Yang, Hailin Xiong, Wei Ye

**Affiliations:** 1 Department of Burn Surgery, Huizhou Municipal Central Hospital, Huizhou, 516001 China; 2 Department of Oncology, Huizhou Municipal Central Hospital, No.41, Erling North Road, Huizhou, 516001 China; 3 Department of Burn Surgery, The First Clinical Medical College of Guangdong Medical University, Huizhou, 516001 China

**Keywords:** Proliferation, LINC00460, ELAVL1, Cutaneous squamous cell carcinoma, Migration, Invasion

## Abstract

Long intergenic noncoding ribonucleic acid (lncRNA) 460 is reportedly associated with carcinogenesis and progression in various types of cancer. However, the mechanisms underlying its action in cutaneous squamous cell carcinoma (CSCC) remain unclear. LINC00460 mRNA expression was analysed using data from the Cancer Genome Atlas (TCGA) and Gene Expression Omnibus (GEO) databases. Cell growth, migration, and invasion were evaluated using Cell Counting Kit-8 (CCK-8), 5-ethynyl-2′-deoxyuridine (EdU), transwell migration and invasion assays after inducing LINC00460 knockdown. A xenograft tumour model was used to determine the effects of LINC00460 on tumour growth and metastasis in vivo. To examine the interaction between LINC00460 and ELAVL1, RNA pulldown and RNA immunoprecipitation assays were performed. LINC00460 was found to be significantly upregulated in CSCC tissues and cell lines. Functionally, LINC00460 knockdown inhibited cell proliferation, migration, and invasion in vitro. Consistent with this, when LINC00460 expression decreased, CSCC tumorigenesis and metastasis in vivo were inhibited. Mechanistically, LINC00460 binds to embryonic lethal abnormal vision like RNA binding protein 1 (ELAVL1) and enhances its stability by inhibiting the β-transducin repeats-containing protein (β-TrCP)-mediated ubiquitination of ELAVL1. Moreover, the effect of LINC00460 silencing on the proliferation, migration, and invasion of CSCC cells could be reversed by overexpressing ELAVL1. Our findings demonstrated that LINC00460 plays a critical role in regulating ELAVL1 function. This highlights the potential targets for the clinical diagnosis and treatment of CSCC.

**Supplementary Information:**

The online version contains supplementary material available at 10.1007/s12033-022-00631-9.

## Introduction

Cutaneous squamous cell carcinoma (CSCC) is the second-most common skin cancer. It develops from premalignant lesions, accounting for approximately 20% of skin-cancer-related mortalities annually [1–4]. Sun exposure, chemical exposure, radiation, exposure to air pollutants, and smoking have been identified as risk factors for CSCC [5]. Although clinical treatments for this type of cancer, including surgery, chemotherapy, and radiotherapy, have progressed in recent times, the prognosis of patients with CSCC remains poor, and their overall survival is unsatisfactory. This is especially true for patients with advanced-stage diseases who have metastatic tumours or are ineligible for surgery [6, 7]. Therefore, the molecular mechanisms involved and novel treatment strategies for CSCC should be urgently identified.

Long non-coding ribonucleic acids (lncRNAs), which are novel non-coding RNAs with limited or no protein-coding ability, have more than 200 nucleotides [8, 9]. Findings from several studies have suggested that lncRNAs play critical roles in biological processes, including proliferation, metastasis, differentiation, and growth [10, 11]. Moreover, the abnormal expression of lncRNAs has been reported to regulate the initiation and progression of different human cancers, including CSCC [12–16]. Recent studies have shown that LINC00460 is overexpressed in multiple cancers and plays an important role in tumour progression, including that in head and neck squamous cell carcinoma, colorectal cancer, osteosarcoma, and hepatocellular carcinoma [17–20]. Data from the Cancer Genome Atlas (TCGA) and Gene Expression Omnibus (GEO) databases showed that LINC00460 was more significantly upregulated in CSCC than in noncancerous tissues. However, the biological behaviour and precise mechanism of action of LINC00460 in CSCC remain poorly defined.

In this study, we investigated the expression pattern and role of LINC00460 in CSCC and the mechanism by which LINC00460 facilitates CSCC development and progression.

## Materials and Methods

### Public Data Analysis

R package was used to analyse the gene expression data of patients with CSCC, which were obtained from the GEO (GSE126209) and TCGA databases. The threshold of |log (fold change) |> 1 and adjusted *P*-value < 0.05 were set as cut-off values to screen differentially expressed genes.

### Cell Culture

Human keratinocytes (HaCaT) and CSCC cells (A431 and HSC-5) were purchased from Jennio Biotech (Guangzhou, China). Cells were cultured in DMEM (Invitrogen, Carlsbad, CA, USA) supplemented with 10% foetal bovine serum (Gibco, Carlsbad, CA, USA), 100 μg/mL streptomycin, and 100 U/mL penicillin. Cells were kept in an incubator at 37 °C with 5% CO_2_.

### Lentivirus and Plasmid Transfection

A short-hairpin RNA (shRNA) for LINC00460 (sh-LINC00460) or corresponding control (sh-NC) lentiviruses were purchased from GenePharma (Shanghai, China) and used to infect CSCC cells treated with 8 mg/mL polybrene for 48 h. Quantitative real-time reverse transcription-polymerase chain reaction (qRT-PCR) was used to evaluate the transduction efficiency of sh-LINC00460. The coding sequences of ELAVL1 were cloned into a pcDNA3.1( +) vector. The vector was then transfected into CSCC cells using Lipofectamine 2000 (Invitrogen).

### qRT-PCR

TRIzol reagent (Invitrogen) was used to isolate total RNA from cells and tissues. Reverse transcription was performed using the PrimeScript RT Reagent Kit (Takara, Dalian, China). The complementary DNA produced was examined by qRT-PCR using Power SYBR Green PCR Master Mix (Applied Biosystems). Relative LINC00460 and ELAVL1 expression levels were calculated using the 2^−ΔΔCt^ method. β-actin was used as an internal reference. The following primers were used: LINC00460: forward 5′- ACGCAGTGGATGAGAACGAA-3′, reverse 5′-GGGGTGACTTCAGAATGCGT-3′; HOXA-AS3: forward 5′-GCTTCCACAATGTCCTGCTTC-3′, reverse 5′-TCAGGCTGCTGGGAAGAGTC-3′; ELAVL1: forward 5′- CCCTCTGGATGGTGGTGAAC-3′, reverse 5′-AAGCGGTTGAGAAAACGCAC-3′; β-actin: forward 5′-TCAGGGAGTAATGGTTGGAAT-3′, reverse 5′- GGTCTCAAACATAATCTGGGTCA-3′.

### Western Blotting

RIPA lysis buffer (Beyotime Biotechnology, Shanghai, China) was used to extract total protein from the cells. Protein concentration was measured using a BCA Kit (Beyotime Biotechnology). The equivalent volumes of protein were subjected to sodium dodecyl sulphate–polyacrylamide gel electrophoresis and transferred to a polyvinylidene fluoride membrane. After blocked by 5% non-fat milk, the membrane was treated overnight with primary antibodies, including those against ELAVL1 (1:1000, Cell Signaling Technology, Danvers, MA, USA) and GAPDH (1:5000; Abcam, Cambridge, MA, USA) at 4 °C. Next, the membrane was treated with a horseradish peroxidase (HRP)-conjugated secondary antibody (1:5000; Abcam) at room temperature for 2 h. Immunoreactive bands were measured using an enhanced chemiluminescence kit.

### Immunohistochemical Staining

Tumour specimens were fixed, dehydrated, and embedded in paraffin. Sections of 4 μm were dewaxed and rehydrated. Following this, the tissue sections were incubated with a Ki67 antibody (1:200, Abcam) and then with a secondary antibody (1:3000, Abcam). Detection was conducted using the DAB Kit (Beyotime Biotechnology) and haematoxylin (Sigma Aldrich).

### Haematoxylin–eosin Staining

Paraffin-embedded lung tissue slices were dewaxed and hydrated. The nuclei were stained with haematoxylin solution (Sigma Aldrich). Following this, cytoplasmic staining was performed using an eosin staining solution (Sigma Aldrich). After the sections were dried, the sheets were preserved using neutral resin.

### Cell Counting Kit-8 (CCK-8) Assay

Cells were seeded in 96-well plates. After 10 μL of the CCK-8 solution (Beyotime Biotechnology) was added to each well at 0, 24, 48, and 72 h, the cells were cultured for 4 h at 37 °C. The absorbance at 450 nm was measured using a microplate reader (Bio-Rad Laboratories, Hercules, CA, USA).

### 5-ethynyl-2′-Deoxyuridine (EdU) Assay

After seeding into a 24-well plate, cells were treated with EdU solution for 2 h. After fixing with 4% paraformaldehyde, the cells were treated with 4′,6-diamidino-2-phenylindole for 30 min. Finally, EdU-positive cells were observed under a microscope (Olympus, Tokyo, Japan).

### Transwell migration and invasion assays

Cells were suspended in a serum-free medium and seeded in the upper chamber of the device with a membrane coated with Matrigel (BD Bioscience, San Jose, CA, USA) for the invasion assay or without Matrigel for the migration assay. Approximately 500 μL of the medium supplemented with 10% foetal bovine serum (as an attractant) was added to the lower chambers. Following a 24-h incubation period, cells on the membrane surface were removed using a cotton swab. Following this, cells in the lower chamber were fixed with 4% paraformaldehyde and stained with 0.1% crystal violet. Migratory/invasive cells were examined under an inverted light microscope (Nikon, Tokyo, Japan).

### RNA Pull-Down Assay

RNA was biotinylated using Biotin-RNA Labelling Mix (Roche Diagnostics International Ltd., Rotkreuz, Switzerland) and transcribed in vitro using T7 RNA polymerase (Roche, Basel, KB, Switzerland). After lysis with a cell lysis buffer, the cells were sonicated and centrifuged. Following this, biotinylated RNA molecules were incubated with a cell supernatant. Subsequently, magnetic beads were used for targeting RNAs, and the co-precipitated protein samples were detected by western blotting.

### RNA Immunoprecipitation Assay

Cells were treated with an RNA immunoprecipitation (RIP) lysis buffer. Next, cell extracts were incubated with magnetic beads conjugated to anti-ELAVL1 antibodies (Cell Signaling Technology) or normal immunoglobulin G (IgG). Proteinase K was used to isolate precipitated RNAs from the magnetic bead complexes. Finally, qRT-PCR was performed to analyse the precipitated RNA [21].

### Mouse Tumour Xenograft Model

A431 cells (1 × 10^7^ cells/mL) infected with sh-LINC00460 or sh-NC lentivirus were subcutaneously inoculated in the axilla of nude mice. The tumour size was estimated every 6 days for 30 days. The tumour volume was calculated using the following formula: V = length × width^2^/2. After the nude mice were euthanised, the weight of xenografts was measured, and tumours from mice were collected for further analysis. sh-NC or sh-LINC00460 stable A431 cells (1 × 10^6^) co-transfected with lentiviruses carrying the luciferase reporter gene were injected into the tail vein of NOD/SCID mice to evaluate the metastatic potential. After 40 days, an in vivo imaging system (IVIS, PerkinElmer, Waltham, MA, USA) was used to assess tumour metastasis. Mice were sacrificed, and their lungs were removed for examination. Metastatic lesions were observed by HE staining. The protocol was approved by the Ethics Committee of Animal Experiments of Huizhou Municipal Central Hospital (No. kyll2021239).

### Cycloheximide-Chase Assay

Cycloheximide (CHX)-chase assay was performed using CHX (Sigma Aldrich), an inhibitor of protein synthesis. Transfected cells in each group were treated with 100 μg/mL CHX. This was followed by measuring the ELAVL1 protein level by western blotting at 0, 3, 6, and 12 h, respectively [22].

### Ubiquitination Assay

The transfected cells were treated with 20 μM MG132 (Sigma Aldrich) for 6 h, which was followed by cell lysis for immunoprecipitation. The cell lysates were incubated overnight with Protein-A/G MagBeads (Yeasen, Shanghai, China) and anti-ELAVL1 antibody (Cell Signaling Technology) or normal rabbit IgG (Cell Signaling Technology) at 4 °C. The protein complexes were retrieved, washed, and subjected to western blotting with an anti-ubiquitin antibody (Cell Signaling Technology) [23].

### Co-Immunoprecipitation Assay

MG132-treated transfected cells were collected and treated overnight with Protein-A/G*.* MagBeads (Yeasen) and anti-ELAVL1 (Cell Signaling Technology) or anti-β-TrCP (Cell Signaling Technology) antibodies at 4 °C. The immune complexes were centrifuged and washed, and the proteins were assessed by western blotting with anti-ELAVL1 or anti-βTrCP antibodies [24].

### Statistical Analysis

Statistical analysis was performed using the Statistical Package for the Social Sciences 21.0 software (SPSS Inc., Chicago, IL, USA). A Student’s *t*-test was performed to analyse comparisons between the two groups. Data are shown as means ± standard deviation (SD). *P <* 0.05 was considered to indicate a statistically significant difference.

## Results

### LINC00460 is Upregulated in CSCC Tissues and Cell Lines

**Fig. 1:**
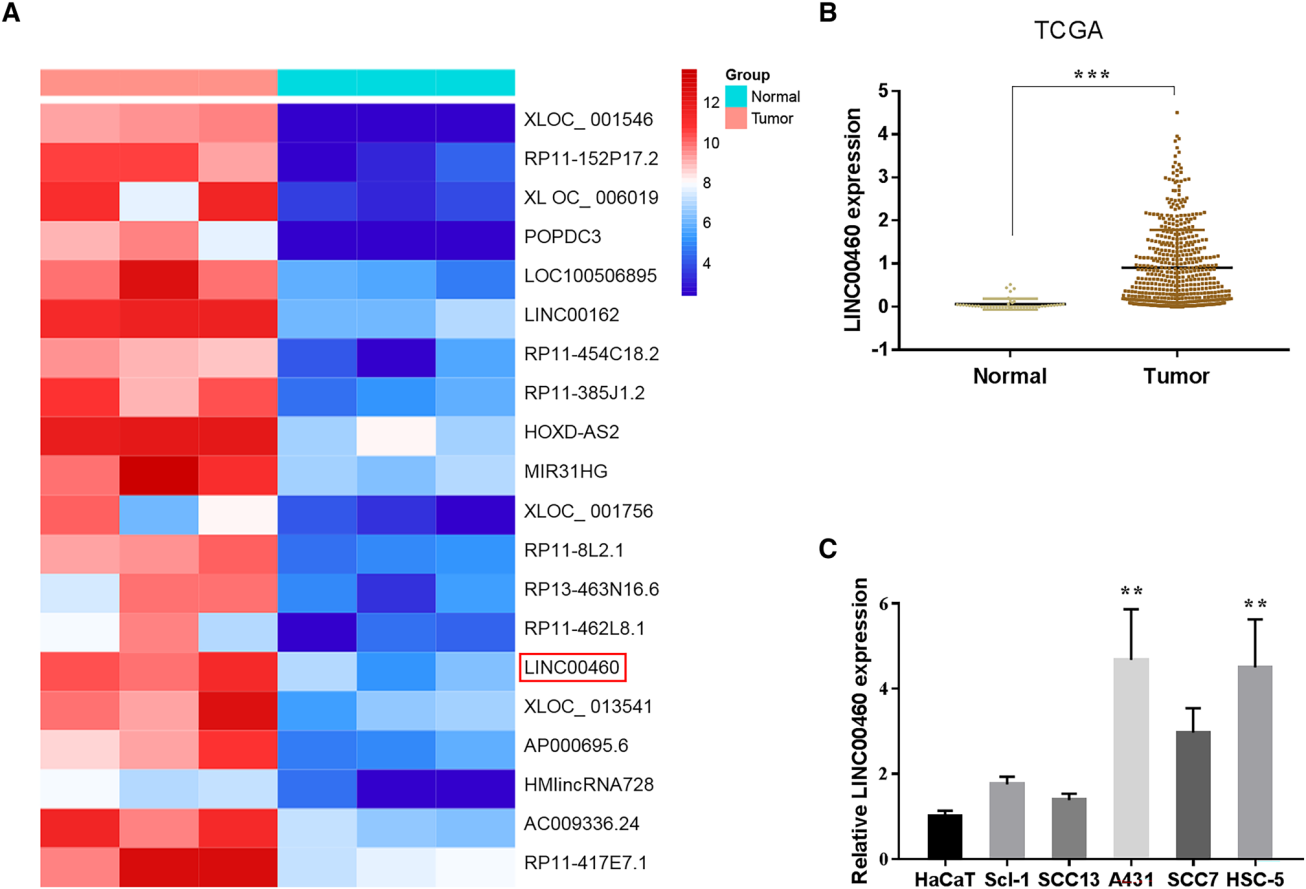
LINC00460 is upregulated in CSCC tissues and cell lines. **a**, **b** LINC00460 upregulation in CSCC, based on data from the GEO and TCGA databases. **c** qRT-PCR to confirm LINC00460 upregulation in CSCC cell lines. Values are presented as mean ± SD from triplicate experiments, *P < 0.05

Ten tumours and nine adjacent normal tissues were used to analyse differentially expressed genes in CSCC based on data from the GEO database (GSE126209). A heatmap showed that the top 20 upregulated lncRNAs in CSCC and LINC00460 were expressed at high levels in patients with CSCC (Fig. 1a). Consistent with this, TCGA data showed a considerably elevated expression of LINC00460 in CSCC tissues (Fig. 1b). qRT-PCR revealed that LINC00460 expression was more significantly enhanced in A431 and HSC-5 cells than in HaCaT cells (Fig. 1c). These results suggested that LINC00460 may play a vital role in CSCC.

### LINC00460 Knockdown Inhibits Cell Proliferation, Migration, and Invasion

**Fig. 2:**
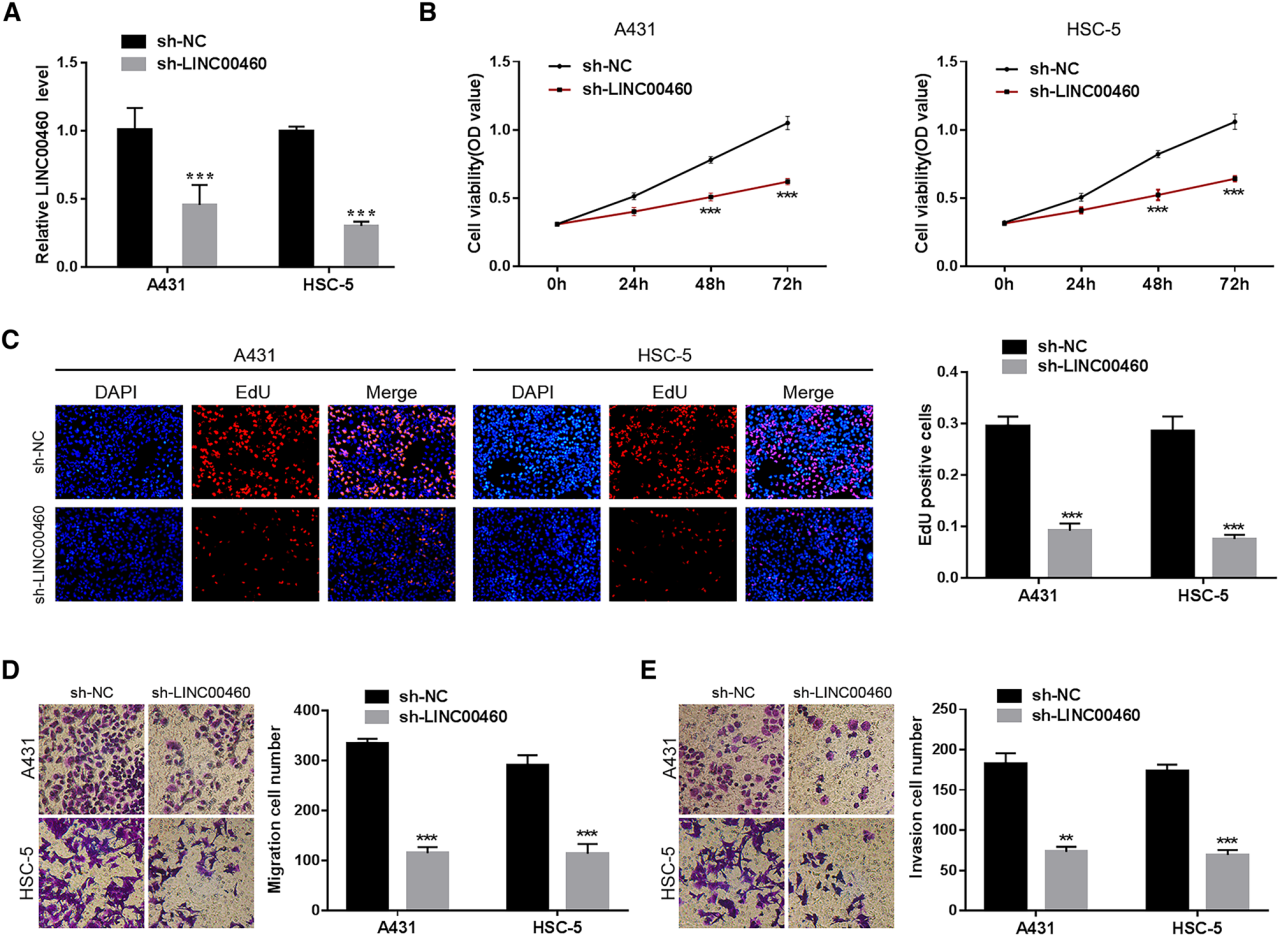
LINC00460 knockdown inhibits cell proliferation, migration, and invasion. **a** LINC00460 expression in A431 and HSC-5 cells infected with sh-LINC00460 or sh-NC lentivirus. **b**, **c** Cell proliferation was evaluated using CCK-8 and EdU assays. **d**, **e** The cell migration and invasion abilities were assessed using transwell migration and invasion assays. Values are presented as the mean ± SD of triplicate experiments, *P < 0.05

Based on the above results, we explored the functions of LINC00460 in CSCC using loss-of-function experiments. First, we infected A431 and HSC-5 cells with sh-LINC00460 or sh-NC lentiviruses to knock down LINC00460 expression. qRT-PCR was used to confirm the knockdown efficiency (Fig. 2a). Findings from the CCK-8 assay indicated that LINC00460 knockdown significantly limited the cell viability (Fig. 2b). Consistent with results from the CCK-8 assay, LINC00460 silencing suppressed the proliferation of A431 and HSC-5 cells in the EdU assay (Fig. 2c). Furthermore, the migratory potential of A431 and HSC-5 cells was inhibited in response to LINC00460 knockdown (Fig. 2d). Likewise, LINC00460 silencing hindered the invasive capacity of A431 and HSC-5 cells (Fig. 2e). These findings suggest that LINC00460 acts as a tumour promoter in CSCC cells.

### Interference with LINC00460 Suppresses Xenograft Tumour Growth and Metastasis* in vivo*

**Fig. 3:**
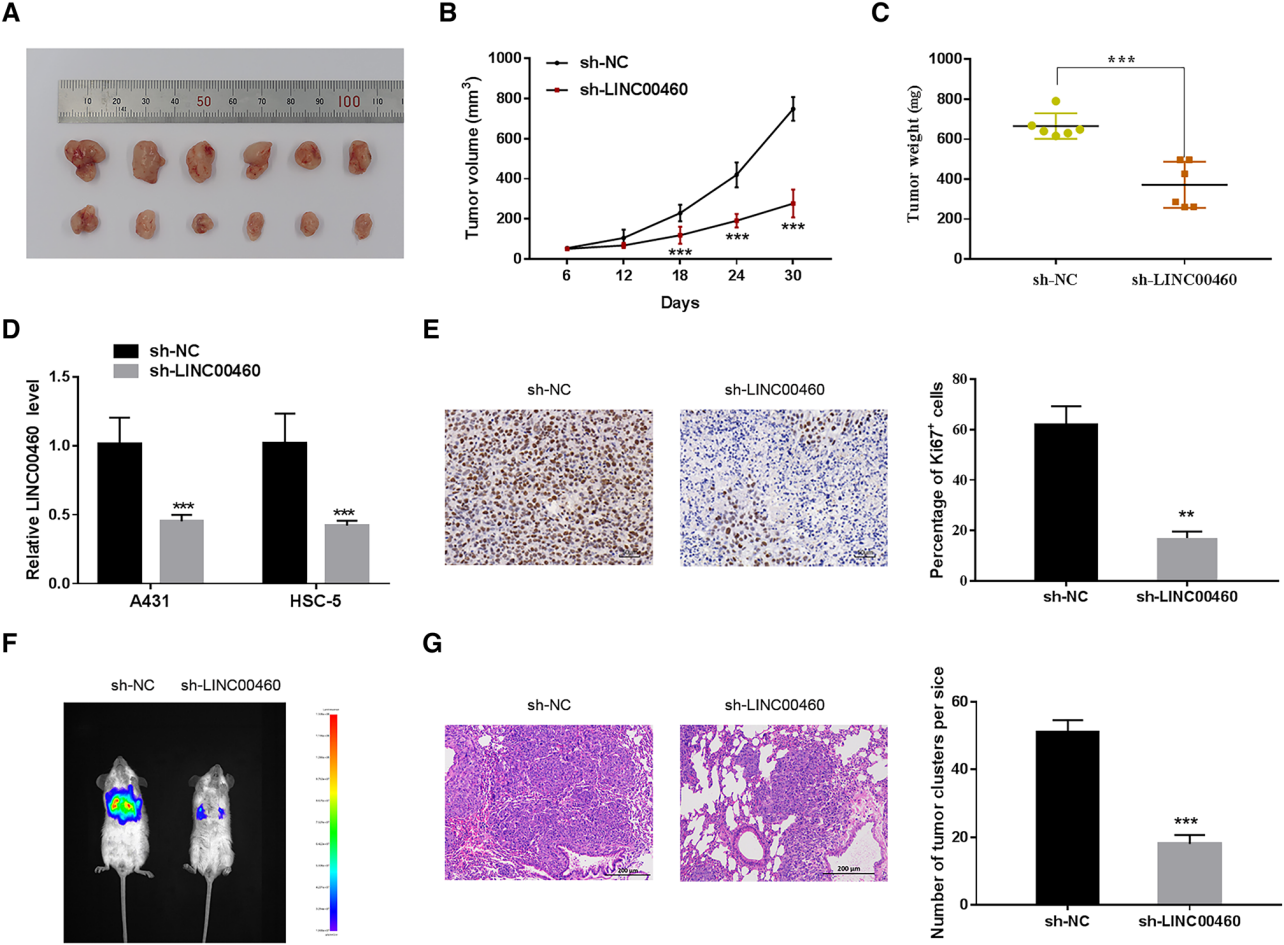
Interference of LINC00460 expression suppresses xenograft tumour growth and metastasis in vivo. **a** representative image of the indicated xenograft tumours. **b** Tumour growth curve. **c** Calculated weight of the indicated xenograft tumours. **d** LINC00460 expression in the indicated xenograft tumour tissues was determined using qRT-PCR. **e** Immunohistochemical staining was performed to measure Ki67 expression. **f** A431 cells stably expressing sh-NC-luciferase or sh-LINC00460-luciferase were injected into the tail veins of NOD/SCID mice. Tumour metastases were imaged using IVIS. **g** Representative haematoxylin–eosin staining images (left) and quantified values (right) of the lung metastatic nodules in different groups. Values are presented as the mean ± SD from six independent experiments, *P < 0.05

The bio-functions of LINC00460 in CSCC in vivo were further explored. First, sh-LINC00460-infected A431 cells were subcutaneously injected into nude mice. The tumour size was more significantly reduced in the LINC00460 knockdown group than in the control group (Fig. 3a). Consistent with our in vitro observations, the tumour volume and weight in the LINC00460-silenced group were significantly lower than those in the control group (Fig. 3b, c). As expected, LINC00460 knockdown decreased the LINC00460 level in xenograft tumour tissues (Fig. 3d). Furthermore, Ki-67 staining of tumour tissues confirmed that the percentage of Ki-67-stained cells in the LINC00460 shRNA-treated group was considerably less than that in the controls (Fig. 3e). Next, sh-LINC00460-infected A431 cells were injected into mice via their tail vein, and the number of lung nodules was determined to assess the anti-metastatic effects of LINC00460 knockdown. As shown in Fig. 3f and g, the number of lung metastatic nodules in the LINC00460 knockdown group was significantly lower than that in the control group. Collectively, these data suggest that LINC00460 ablation could block the growth and metastasis of CSCC cells in vivo.

### LINC00460 Interacts with ELAVL1 and Inhibits ELAVL1 Degradation

**Fig. 4:**
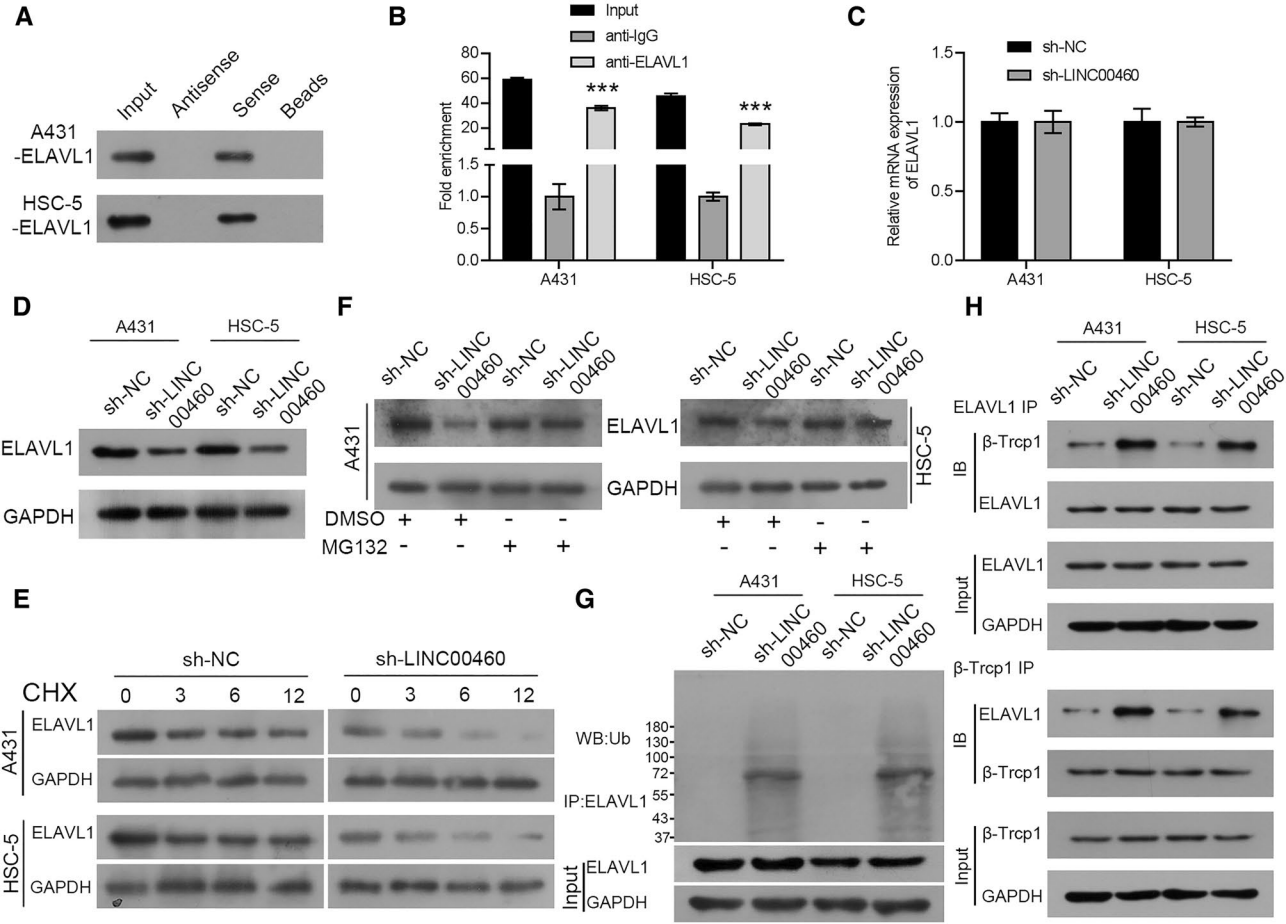
LINC00460 interacts with ELAVL1 and inhibits ELAVL1 degradation. **a** The specific interaction of LINC00460 with ELAVL1. **b** RIP assay shows the relationship between ELAVL1 and LINC00460 expression. **c**, **d** ELAVL1 mRNA and protein expression levels in A431 and HSC-5 cells infected with sh-LINC00460 or sh-NC lentiviruses, as determined by qRT-PCR and western blotting. **e** Western blotting analysis of ELAVL1 in transfected A431 and HSC-5 cells treated with cycloheximide (CHX) for the indicated duration. **f** ELAVL1 protein level in transfected A431 and HSC-5 cells treated with MG132 for the indicated duration. **g** Western blotting images showing ELAVL1-associated ubiquitination in LINC00460 knockdown cells. **h** CO-immunoprecipitation to assess the interaction between ELAVL and β-Trcp1 in LINC00460 knockdown cells treated with MG132. Values are presented as the mean ± SD from triplicate experiments, *P < 0.05

We further investigated the molecular mechanism of action of LINC00460 in CSCC. Findings from previous reports have revealed that the lncRNA could function in combination with proteins [25, 26]. Bioinformatics databases (ENCORI and RBPDB) were used to identify proteins associated with LINC00460, and a potential interaction between LINC00460 and ELAVL1 was predicted. Thereafter, we performed an RNA pull-down assay and found that LINC00460 interacts with ELAVL1 in CSCC cells (Fig. 4a). Moreover, findings from the RIP assay confirmed that ELAVL1 is directly bound to LINC00460, and a higher LINC00460 enrichment was observed with anti-ELAVL1 antibodies than with IgG (Fig. 4b). Furthermore, the depletion of LINC00460 reduced the protein expression of ELAVL1 but not the mRNA expression. Thus, LINC00460 may regulate the protein expression of ELAVL1 at the post-translational level (Fig. 4c, d). To test this hypothesis, we evaluated the effect of LINC00460 on the stability of ELAVL1 protein by treating CSCC cells with CHX to suppress protein synthesis. As shown in Fig. 4e, ELAVL protein expression declined persistently in LINC00460 knockdown CSCC cells in the presence of CHX, implying that the silencing of LINC00460 promoted the degradation of ELAVL. We then validated whether LINC00460 knockdown affected the stability of ELAVL via proteasome-mediated degradation. As shown in Fig. 4f, treatment with the proteasome inhibitor MG132 resulted in ELAVL accumulation in LINC00460 knockdown CSCC cells. Besides, LINC00460 silencing-induced ELAVL ubiquitination (Fig. 4g). A previous study showed that β-Trcp1 acts as an E3 ligase for ELAVL ubiquitination and degradation [24]. Here, we performed CO-immunoprecipitation experiments and found that LINC00460 inhibition promoted the interaction between ELAVL and β-Trcp1 in A431 and HSC-5 cells (Fig. 4h). Collectively, these data indicate that LINC00460 enhances ELAVL protein expression by suppressing β-Trcp1-mediated ELAVL ubiquitination and degradation.

### LINC00460 enhances CSCC Cell Proliferation, Migration, and Invasion via ELAVL1 Expression

**Fig. 5:**
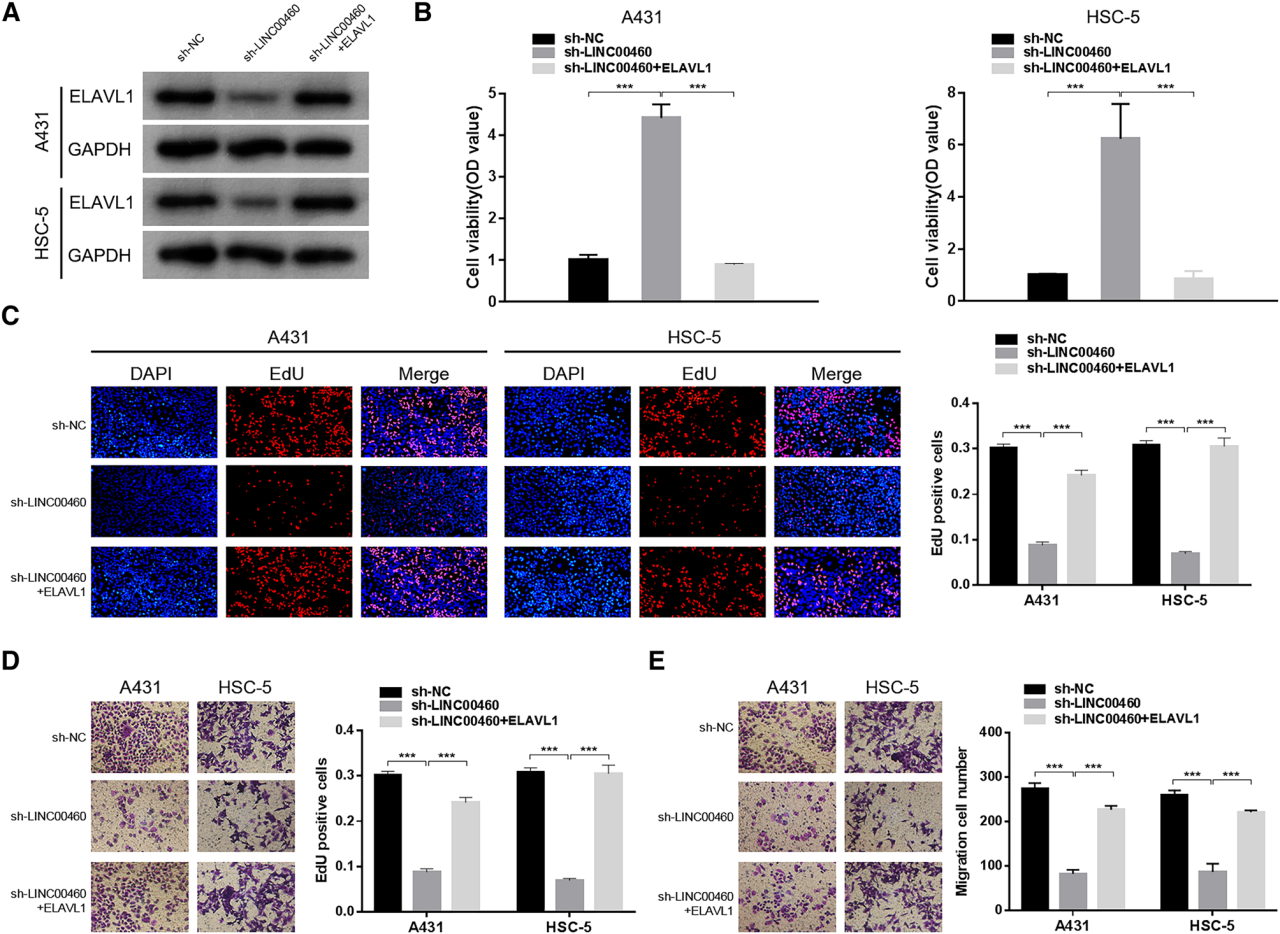
LINC00460 enhances CSCC cell proliferation, migration, and invasion via ELAVL1 expression. **a** ELAVL1 protein expression measured in A431 and HSC-5 cells co-transfected with sh-LINC00460 lentivirus and ELAVL1 plasmid using qRT-PCR and western blotting, respectively. **b**, **c** Cell proliferation was assessed via CCK-8 and EdU analysis. **d**, **e** Cell migration and invasion ability were assessed using transwell migration and invasion assays. Values are presented as the mean ± SD from triplicate experiments, *P < 0.05

To determine whether LINC00460 accelerates CSCC cell function by regulating ELAVL1 expression, we co-transfected A431 and HSC-5 cells with the sh-LINC00460 lentivirus and ELAVL1 plasmid. As indicated by the results of western blotting experiments, LINC00460 knockdown-induced ELAVL1 protein expression inhibition was abrogated in response to ELAVL1 overexpression (Fig. 5a). The inhibition of cell proliferation by LINC00460 silencing was reversed upon ELAVL1 overexpression, as determined in the CCK-8 and EdU assays (Fig. 5b, c). Consistent with this, ELAVL1 overexpression also reversed the suppression of cell migration and invasion induced upon LINC00460 knockdown (Fig. 5d, e). Collectively, these findings indicate that LINC00460 is dependent on ELAVL1 to enhance CSCC cell growth, migration, and invasion.

## Discussion

In the present study, LINC00460 expression was higher in CSCC tissues than in adjacent tissues. To the best of our knowledge, this study is the first to determine LINC00460 function in CSCC. Loss-of-function assays showed that LINC00460 knockdown inhibited CSCC cell growth, invasion, and migration. In vivo, LINC00460 silencing significantly decreased tumour growth and metastasis. Mechanistically, LINC00460 promoted CSCC progression by binding to ELAVL1 and regulating ELAVL1 stability. Collectively, our findings confirmed that LINC00460 serves as a novel oncogene in CSCC.

LINC00460 has been identified as an oncogene in various tumours [27–30]. For instance, Wang et al. showed that LINC00460 was significantly upregulated in patients with colorectal cancer, and its expression was correlated with the clinical stage, pathological differentiation, and survival rate of patients [28]. Feng et al. reported that LINC00460 was markedly upregulated in papillary thyroid carcinoma tissues and cell lines and suppressed cell proliferation, migration, and invasion [29]. Moreover, Han et al. showed that LINC00460 exerted anti-tumour effects in hepatocellular carcinoma in vitro and in vivo through the miR-342-3p/AGR2 axis [30]. In the present study, LINC00460 expression was significantly enhanced in CSCC tissues and cell lines. In the in vitro and in vivo experiments, LINC00460 depletion suppressed CSCC cell viability, invasiveness, and migratory potential and tumour growth and metastasis. These findings suggest that LINC00460 plays a carcinogenic role in CSCC progression.

LncRNAs regulate the expression levels of target proteins by modulating their stability [31, 32]. For example, LncRNA FAM83H-AS1 enhanced the stabilization of ELAVL1 protein by interacting with ELAVL1 [32]. In our study, ELAVL1 was found to be the downstream protein of LINC00460 in RNA pull-down and RIP assays. Furthermore, LINC00460 stabilized ELAVL1 to prevent its degradation via ubiquitination. ELAVL1 has been identified as an oncogenic gene in various cancers, and its expression is closely associated with the progression of cancer [33–36]. A previous study demonstrated that ELAVL1 promotes breast cancer proliferation, metastasis, and chemoresistance [33]. Moreover, Hu et al. showed that ELAVL1 facilitates cell proliferation and represses apoptosis in nasopharyngeal carcinoma [34]. In addition, Long et al. revealed that ELAVL1 accelerated adrenocortical carcinoma cell growth both in vivo and in vitro [35]. Consistent with these findings, we observed that ELAVL1 overexpression could partially counteract the decline in cell proliferation, migration, and invasion induced by LINC00460 knockdown.

Collectively, our findings revealed that LINC00460 promotes CSCC cell proliferation, migration, and invasion by stabilizing the ELAVL1 protein. The findings of this study provide novel insights into the mechanisms underlying CSCC, and LINC00460 might serve as a potential novel diagnostic and therapeutic target in rectal cancer treatment. However, further investigations are warranted to evaluate the molecular mechanism of the LINC00460/ELAVL1 axis in regulating CSCC progression.

## Supplementary Information

Below is the link to the electronic supplementary material.

Supplementary file1 (TIF 12346 kb)

Supplementary file2 (TIF 6007 kb)

Supplementary file3 (TIF 5851 kb)

Supplementary file4 (TIF 11993 kb)

Supplementary file5 (TIF 7969 kb)

Supplementary file6 (TIF 6247 kb)

## Data Availability

The data that support the findings of this study are available from the corresponding author upon reasonable request.
