# Supplementary figures and images for: LINC00460 Promotes Cutaneous Squamous Cell Carcinoma Progression Through Stabilizing ELAVL1 Protein
Source: Mol Biotechnol. 2022 Dec 13;65(8):1296–305. doi: 10.1007/s12033-022-00631-9 (PMC10352421; doi:10.1007/s12033-022-00631-9)

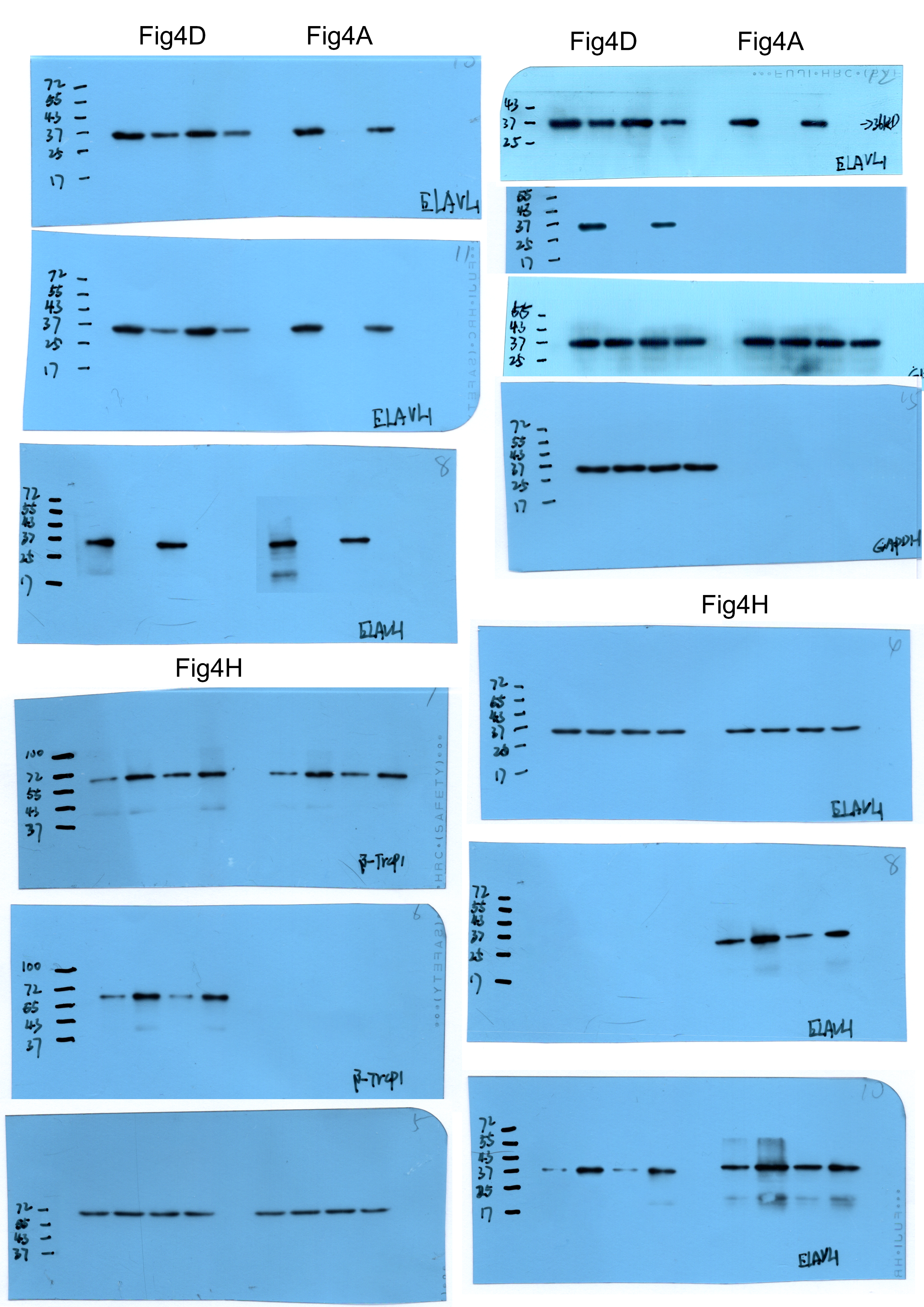

Supplement: Supplementary file 1 — Supplementary file1 (TIF 12346 kb) [file 12033_2022_631_MOESM1_ESM.tif]

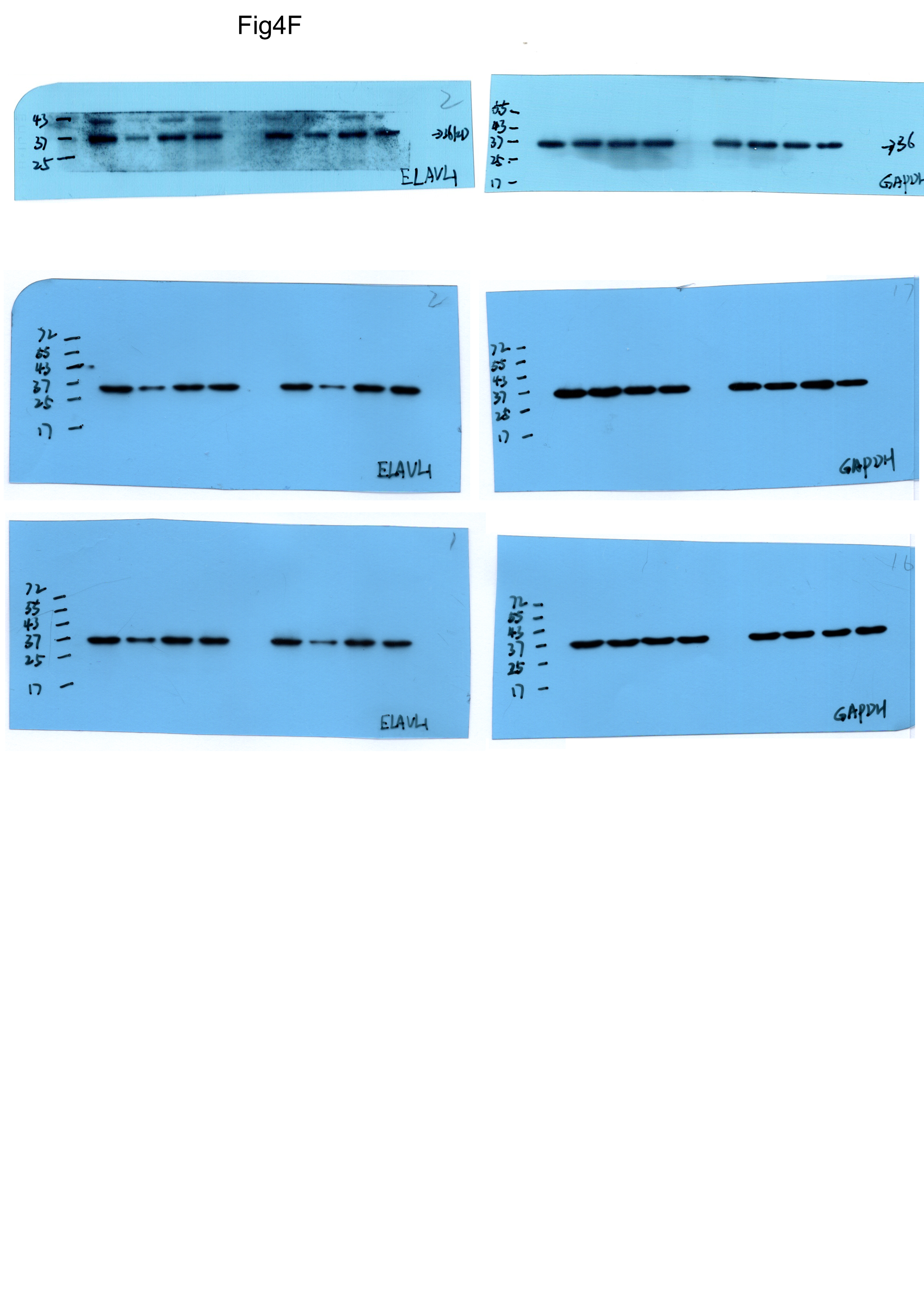

Supplement: Supplementary file 2 — Supplementary file2 (TIF 6007 kb) [file 12033_2022_631_MOESM2_ESM.tif]

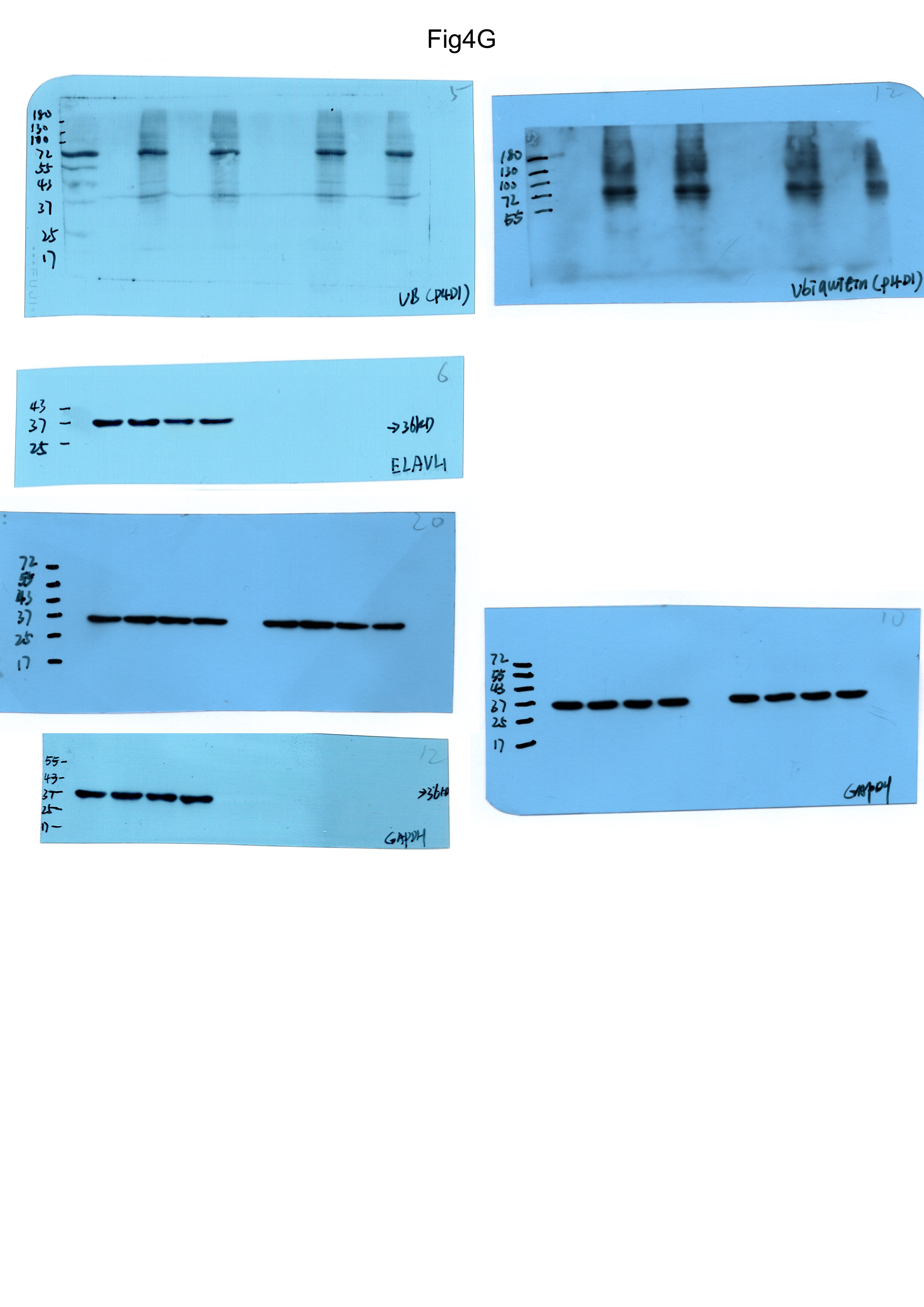

Supplement: Supplementary file 3 — Supplementary file3 (TIF 5851 kb) [file 12033_2022_631_MOESM3_ESM.tif]

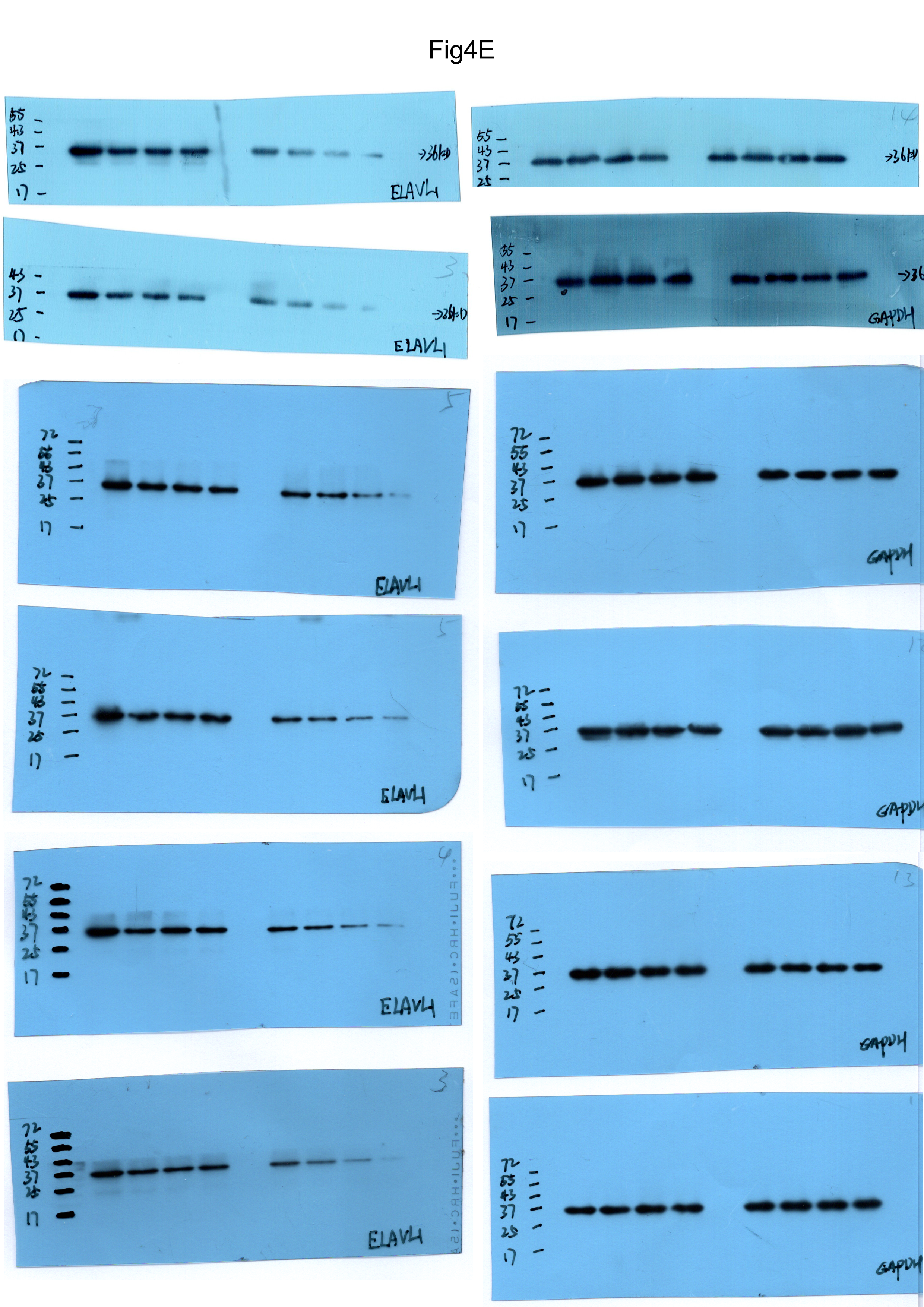

Supplement: Supplementary file 4 — Supplementary file4 (TIF 11993 kb) [file 12033_2022_631_MOESM4_ESM.tif]

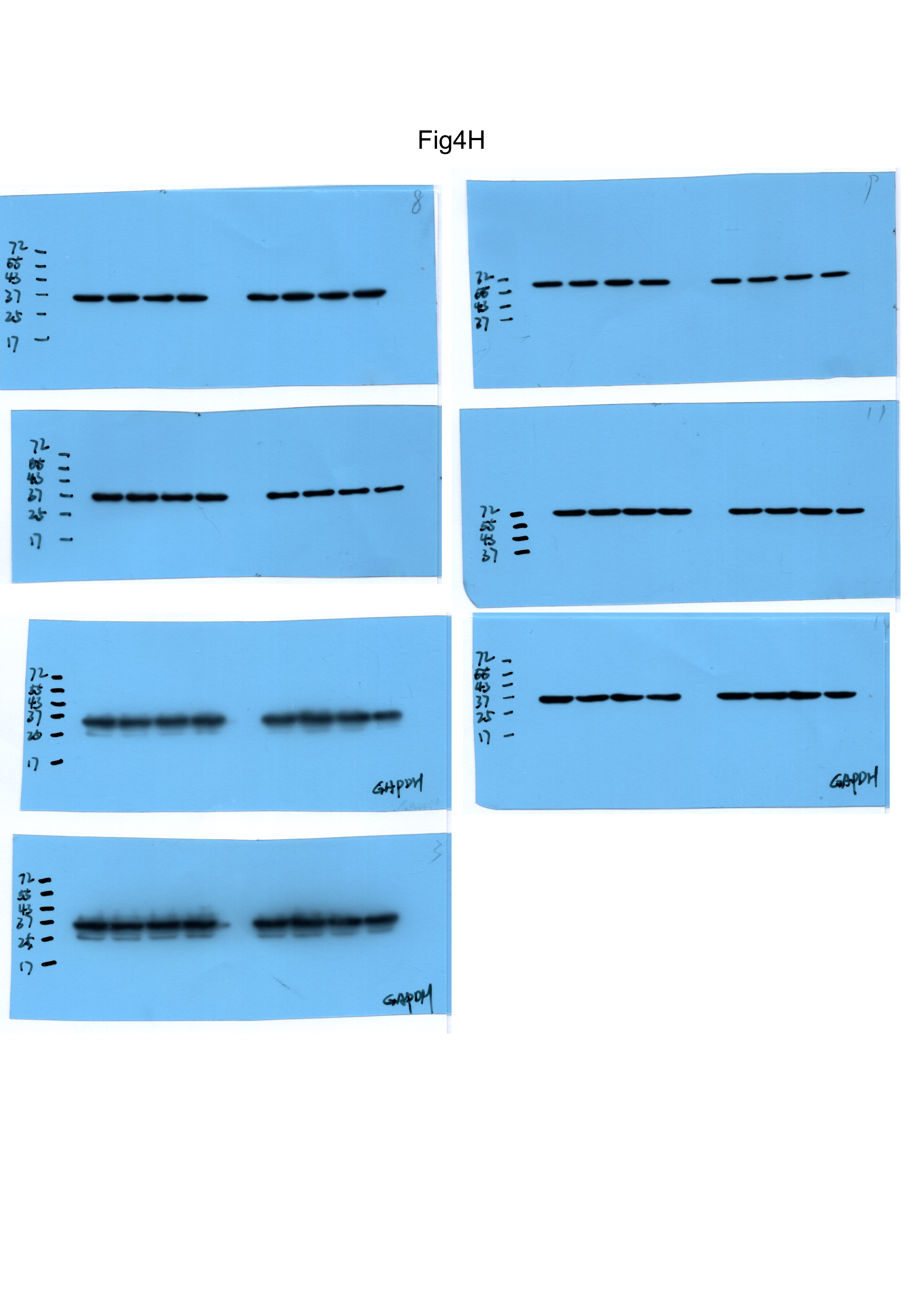

Supplement: Supplementary file 5 — Supplementary file5 (TIF 7969 kb) [file 12033_2022_631_MOESM5_ESM.tif]

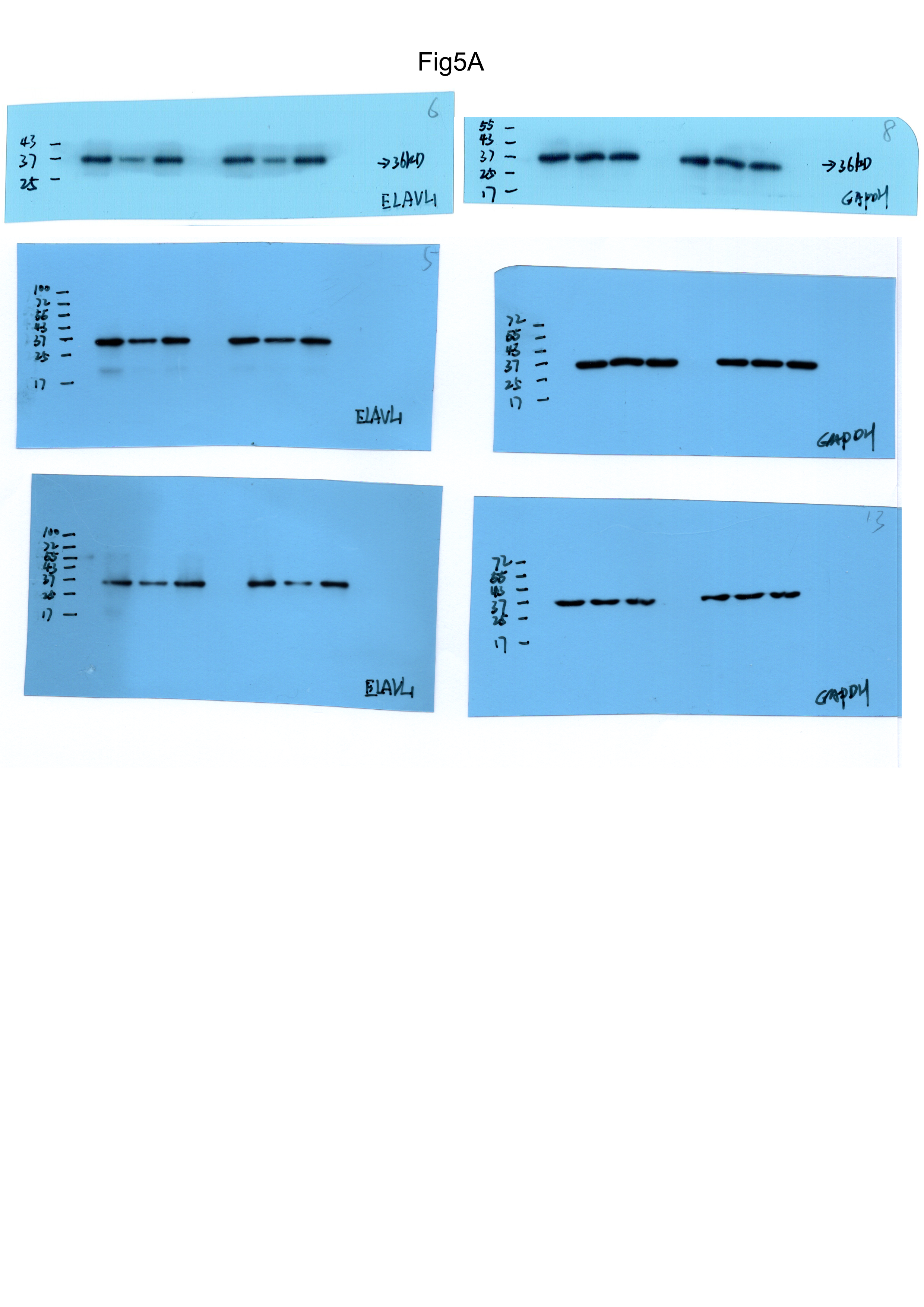

Supplement: Supplementary file 6 — Supplementary file6 (TIF 6247 kb) [file 12033_2022_631_MOESM6_ESM.tif]
